# Is there different risk of cancer among end‐stage renal disease patients undergoing hemodialysis and peritoneal dialysis?

**DOI:** 10.1002/cam4.1289

**Published:** 2018-01-22

**Authors:** Yi‐Che Lee, Shih‐Yuan Hung, Hao‐Kuang Wang, Chi‐Wei Lin, Hsi‐Hao Wang, Min‐Yu Chang, Junne‐Ming Sung, Yuan‐Yow Chiou, Sheng‐Hsiang Lin

**Affiliations:** ^1^ Division of Nephrology Department of Internal Medicine E‐DA Dachang Hospital/ I‐Shou University Kaohsiung Taiwan; ^2^ School of Medicine for International Students E‐DA Hospital/ I‐Shou University Kaohsiung Taiwan; ^3^ Department of Neurosurgery E‐DA Hospital/ I‐Shou University Kaohsiung Taiwan; ^4^ Department of Medical Education E‐DA Hospital/ I‐Shou University Kaohsiung Taiwan; ^5^ Division of Nephrology Department of Internal Medicine National Cheng Kung University Hospital Tainan Taiwan; ^6^ Department of Pediatrics National Cheng Kung University Hospital Tainan Taiwan; ^7^ Institute of Clinical Medicine College of Medicine National Cheng Kung University Tainan Taiwan; ^8^ Biostatistics Consulting Center National Cheng Kung University Hospital Tainan Taiwan; ^9^ Department of Public Health College of Medicine National Cheng‐Kung University Tainan Taiwan

**Keywords:** Cancer, cohort study, dialysis, end‐stage renal disease, National Health Insurance

## Abstract

Cancer is a global issue in recent decade. Despite this alarming increase in the incidence of cancer, to date, whether the risk of developing cancer differs among peritoneal dialysis (PD) and hemodialysis (HD) patients is still uncertain. In this retrospective cohort study, data were obtained from the National Health Insurance Research Database of Taiwan, which provides coverage to almost 99% of the nation's population. After matching, a total of 4491 (or 3369) incident PD patients and 8982 (or 6738) incident HD patients between 2000 and 2009 were enrolled from the database. In addition, 22,455 (or 16,845) nondialysis patients were selected as a control group. The patients were monitored for the occurrence of cancer until 2010, and their data were analyzed using several different models. In general, the results showed that the risks of hepatocellular, kidney, bladder, extra kidney/bladder urinary tract, and thyroid cancers were higher in dialysis patients. We also compared the risk of cancer between two dialysis groups by using the HD patients as the reference group. The result showed that there is no significant different for each cancer risk between two dialysis groups. In conclusion, dialysis patients had a higher risk of certain types of cancer than those in the nonuremia group. However, there was no significant difference in the cancer risk between the two dialysis groups when compared directly.

## Introduction

End‐stage renal disease (ESRD) is an important public health problem worldwide. In the United States, it is estimated that more than 2 million people will require renal replacement therapy by the year 2030 1. In Taiwan, the rapidly increasing number of uremic patients has also placed a substantial burden on health care resources 2.

Cancer is a leading cause of death worldwide in recent decades and it is a major health problem around the globe.

Despite this alarming increase in the incidence of cancer, to date, the correlation of ESRD and cancer is still uncertain. Although many studies have described a relationship between ESRD and cancer 3, 4, 5, 6, 7, 8, 9, 10, 11, 12, 13, 14, 15, 16, 17, 18, 19, 20, 21, most of these studies have been limited by a noncohort study design, enrolling a relatively small number of patients and lacking an appropriate comparison group, or a lack of adequate potential confounders in the regression model. Besides, death may act as a competing risk with cancer event, but these studies did not use competing risk models. Most importantly, we have reasonable grounds to suspect that hemodialysis (HD) and peritoneal dialysis (PD) patients may have different cancer risks. In patients undergoing PD therapy, the conventional bioincompatible dialysate, which is hypertonic, has a high glucose content, an acidic pH, and contains both lactate and glucose degradation products (GDPs), induces peritoneal damage and chronic inflammation 22, 23, 24. It is known that chronic inflammation is one of the risk factors for cancer 25. By contrast, HD patients have higher hepatitis B and C infection risks, and chronic hepatitis B and C are risk factors for hepatocellular carcinoma 26. A previous study also found that HD patients had a higher risk of peptic ulcer disease (PUD) than PD patients, and PUD has been proven to have a high correlation with *Helicobacter pylori* infection 27. *Helicobacter pylori* is definitely carcinogenic in the case of gastric cancer 28. Therefore, we suspect that HD and PD patients may have different cancer risks, especially with regard to malignancy of the intra‐abdominal organs. However, data concerning differences in cancer risk among ESRD patients receiving HD and PD are still limited. Therefore, the aim of this study was to investigate the important issue of cancer risk in ESRD patients and whether patients who received dialysis through different modalities had different risks for cancer in a large‐scale population‐based cohort study.

## Materials and Methods

This study was designed as a population‐based retrospective cohort study and the data were obtained from the Taiwan National Health Insurance (NHI). New onset HD patients and PD patients within a defined period were enrolled as the HD or PD cohort. In the same period, non‐ESRD individuals were also enrolled as a comparison cohort. We then monitored the cancer event in these groups over time.

### Ethics statement

The study was approved by the ethics committee/Institutional Review Board of National Cheng Kung University Hospital (IRB number: A‐EX‐103‐026).

### Database

The Taiwan NHI is a mandatory social health insurance plan that started in 1995. Almost 99% of Taiwan's population of 23 million is enrolled in this plan. The Taiwan NHI Research Database (NHIRD) has been used for epidemiological studies and for studying information on prescription drug use, among other purposes. The accuracy of major disease diagnoses recorded in the NHIRD has been validated, and the recorded data have been shown to be of high quality 27, 29, 30, 31, 32, 33, 34, 35, 36.

### Dialysis cohort

**Figure 1:**
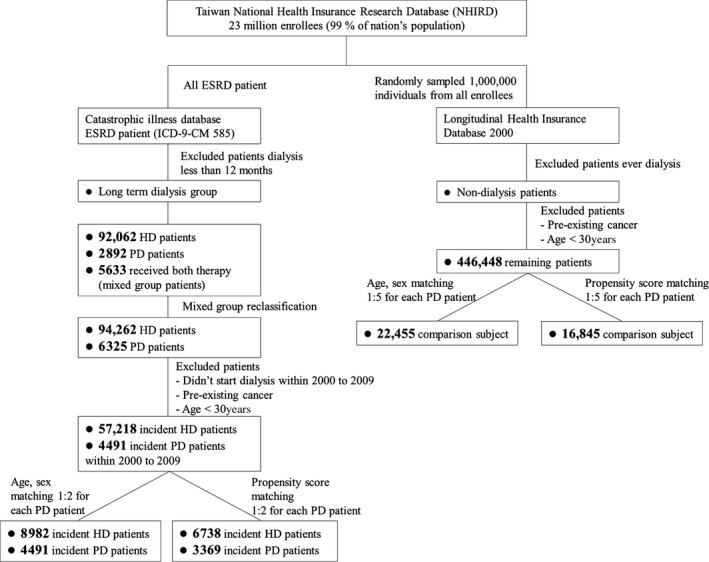
Study flowchart. ESRD, end‐stage renal disease; HD, hemodialysis; ICD‐9‐CM, International Classification of Diseases, Ninth Revision, Clinical Modification; mixed group, patients ever receive both HD and PD therapy; PD, peritoneal dialysis.

All disease diagnosis codes were assigned according to the International Classification of Diseases, Ninth Revision, Clinical Modification (ICD‐9‐CM). First, the incident ESRD patients (who have ICD‐9‐CM code 585 and a catastrophic illness card for ESRD) who started receiving dialysis between 1 January 2000 and 31 December 2009 were enrolled as the dialysis cohort (Fig. 1). In Taiwan, if patients are diagnosed as having a disease classified as a catastrophic illness by the Ministry of Health and Welfare, the patient can apply for a catastrophic illness certificate 37. The application will be formally reviewed according disease information. Then the patients with catastrophic illness certificate do not need to pay a copayment for outpatient or inpatient care for the related illness. For example, all uremic patients are qualified by NHI to apply for catastrophic illness certificate about ERSD, and if approved, these patients do not have copayments for their dialysis treatment. Patients who did not receive dialysis for more than 1 year were excluded. Patients who had a history of cancer before enrollment were also excluded. We enrolled people older than 30 years. These ESRD populations were then divided into HD and PD groups according to their dialysis modality. If patients had ever received both HD and PD in their lifetime, then they were classified as HD if the HD duration was 6 months longer than the duration of PD, and vice versus for the PD group. Finally, PD and HD patients were enrolled in the ratio 1:2 in an age/sex‐matched model and a propensity score‐matched model. All individuals were followed up until a cancer diagnosis, receipt of a renal transplant, death, or the end of the study (2010).

### Comparison cohort

According to Taiwan's NHI report, in 2000, there were about 23 million enrollees (99% of the nation's population). Among these enrollees, 1,000,000 people were randomly selected, and all data of the sample group constitute the Longitudinal Health Insurance Database (LHID) 2000 38. No significant differences in age, sex, or health care costs were found between the LHID 2000 sample group and all enrollees. LHID 2000 has been used for many research purposes and the data have been shown to be of high quality 36. Our comparison cohort was selected from LHID 2000. First, patients who previously underwent dialysis were excluded. To ensure comparability, we also excluded those younger than 30 years and those with an existing cancer history before enrollment. Finally, the comparison group was randomly selected from the remaining patients at a ratio of 1:5 with PD patients in an age/sex‐matched model and a propensity score‐matched model. All individuals were followed up until a cancer diagnosis, receipt of a renal transplant, death, or the end of the study (2010).

### Matching

This study used two different matching models to ensure comparability in different baseline characteristics. First, frequency matching for sex, age, and year of receiving medical care to ensure comparability in these factors was performed. Second, propensity score matching was performed by using a greedy algorithm to minimize potential selection bias in different cohorts. The propensity score is defined as the likelihood of choosing PD as their first dialysis choice, given all covariates. For each patient, an estimated propensity score was calculated using the logistic regression method to evaluate the differences in the baseline characteristics and comorbidities. The covariates in the propensity score model included 17 items, including sex, age, diabetes mellitus (DM), hypertension (HTN), congestive heart failure (CHF), hyperlipidemia, chronic hepatitis and liver cirrhosis, autoimmune disease, gout, coronary artery disease (CAD), cerebrovascular disease, atrial fibrillation (Af), dementia, peripheral atherosclerosis, chronic lung disease, depression, and alcohol‐related illness. The c‐statistic values for the propensity score models were 0.931 (PD vs. comparison) and 0.774 (PD vs. HD).

### Potential confounders

We identified potential confounding risk factors for cancer for individuals in all three groups. These risk factors included DM, hyperlipidemia, HTN, CHF, CAD, Af, cerebrovascular disease, chronic lung disease, autoimmune disease, chronic hepatitis and liver cirrhosis, depression, dementia, and alcohol‐related illness. The prescription of medications that could confound the cancer risk were also identified, such as insulin, oral antidiabetic agents, statins, fibrate, aspirin, nonsteroidal anti‐inflammatory drugs (NSAIDs), angiotensin‐converting enzyme inhibitor (ACEI), and hormone replacement therapy. Comorbidities and medication were considered only 1 year before and after the index date.

### Main outcome measure

The endpoint of the study was the occurrence of any of the following malignancies (based on catastrophic illness registration cards for each cancer): lip, oral cavity, and pharynx cancer (ICD‐9‐CM code 140–149), esophageal cancer (150), gastric cancer (151), colorectal cancer (153–154), hepatocellular carcinoma (155.0), gallbladder and bile duct cancer (156, 155.1), pancreatic cancer (157), retroperitoneal and peritoneal cancer (158), lung cancer (162), breast cancer (female) (174), cervical cancer (180), prostate cancer (185), kidney cancer (189.0), bladder cancer (188), extra kidney and bladder urinary tract cancer (189.1, 189.2, 189.3, 189.4, 189.8, 189.9), brain cancer (191), thyroid cancer (193), and lymphatic and hematopoietic cancer (200–208).

### Validation

We validated our method for the identification of ESRD and cancer (catastrophic illness registration cards combined with ICD‐9‐CM codes) by analyzing the medical records (charts) of 100 patients in E‐DA Hospital, an 1100 bed teaching hospital in Taiwan. We randomly selected 50 patients who had catastrophic illness registration cards for ESRD and 50 patients who had catastrophic illness registration cards for different cancers from the patient claims database between 2008 and 2010 for the hospital. Positive predictive values of ESRD and cancer were estimated. The results showed a positive predictive value of 100% for both ESRD and cancer.

### Statistical analysis

Baseline descriptive data of enrollees were presented as the mean ± SD for frequency and as continuous variables and percentage for categorical variables. Pearson chi‐square tests, one‐way analysis of variance, and standardized differences were used to analogize the clinical characteristics between the three cohorts. To investigate the impact of the two different dialysis modalities on cancer risk, multivariable Cox proportional hazard models were used. Survival time was censored if the patient received a renal transplant, died, or reached the end of the study period. Because death may act as a competing risk for cancer, competing risk models were also used to adjust for risk of death (R package “cmprsk”) 39. In this study, all statistical analyses were conducted with SAS 9.3 statistical software (SAS Institute Inc., Cary, NC) and R statistical software, version 3.0.3 (R Foundation for Statistical Computing).

## Results

As Figure 1 shows, we enrolled all dialysis patients in our country initially. After exclusion, there were a total of 35,928 subjects enrolled, including 8982 incident HD patients, 4491 incident PD patients, and 22,455 nondialysis patients in the age‐ and sex‐matched models. A total of 26,952 subjects were enrolled, including 6738 incident HD patients, 3369 incident PD patients, and 16,845 nondialysis patients in the propensity score‐matched model.

Table 1 shows the demographic characteristics and clinical comorbidities of the three cohorts in the age‐ and sex‐matched models and the propensity score‐matched model. In the age‐ and sex‐matched models, the dialysis patients had significantly higher incidence rates of most comorbidities than the comparison cohort, including DM, hyperlipidemia, HTN, CHF, CAD, Af, cerebrovascular disease, chronic hepatitis and liver cirrhosis, and depression. In the propensity score‐matched model, by contrast, the incidence rates of most comorbidities were similar, except DM, CHF, and chronic lung disease.

**Table 1:** Demographic characteristics and comorbidities of peritoneal dialysis, hemodialysis, and nondialysis comparison cohorts

Characteristic	Age and sex matched				Propensity score matched			
	PD(*N* = 4491)	HD(*N* = 8982)	Comparison(*N* = 22,455)	*P*	PD(*N* = 3369)	HD(*N* = 6738)	Comparison(*N* = 16,845)	*P*
Age, years								
30–45	1304 (27.6)	2608 (27.6)	6520 (27.6)	1.000	845 (25.1)	1507 (22.4)	3589 (21.3)	<0.001
45–60	1557 (34.5)	3114 (34.5)	7785 (34.5)		1453 (43.1)	2964 (44.0)	7500 (44.5)	
60+	1630 (36.3)	3260 (36.3)	8150 (36.3)		1071 (31.8)	2267 (33.6)	5756 (34.2)	
Mean ± SD	57.5 ± 11.0	58.5 ± 11.7	56.8 ± 11.8	<0.001	54.7 ± 12.9	56.4 ± 13.1	56.7 ± 13.5	<0.001
Male, *n* (%)	2473 (55.1)	4946 (55.1)	12,365 (55.1)	1.000	1496 (44.4)	3145 (46.7)	7671 (45.5)	0.08
Comorbidities, %								
Diabetes mellitus	2509 (55.9)	6604 (73.5)	5302 (23.6)	<0.001	961 (28.5)	2210 (32.8)	4721 (28.0)	<0.001
Hyperlipidemia	2886 (64.3)	4246 (47.3)	6209 (27.7)	<0.001	1782 (52.9)	3649 (54.2)	9035 (53.6)	0.48
Hypertension	3764 (83.8)	7099 (79.0)	9227 (41.1)	<0.001	2885 (85.6)	5894 (87.5)	14,733 (87.5)	0.01
Congestive heart failure	650 (14.5)	2351 (26.2)	1056 (4.7)	<0.001	309 (9.2)	655 (9.7)	1331 (7.9)	<0.001
Coronary artery disease	1169 (26.0)	3729 (41.5)	3701 (16.5)	<0.001	636 (18.9)	1343 (19.9)	3200 (19.0)	0.22
Atrial fibrillation	174 (3.9)	421 (4.7)	474 (2.1)	<0.001	78 (2.3)	149 (2.2)	338 (2.0)	0.39
Cerebrovascular disease	718 (16.0)	2101 (23.4)	2610 (11.6)	<0.001	314 (9.3)	657 (9.8)	1612 (9.6)	0.78
Chronic lung disease	570 (12.7)	1321 (14.7)	3488 (15.5)	<0.001	305 (9.1)	566 (8.4)	1657 (9.8)	<0.001
Autoimmune disease	252 (5.6)	405 (4.5)	1084 (4.8)	0.019	145 (4.3)	286 (4.2)	779 (4.6)	0.38
Chronic hepatitis and liver cirrhosis	706 (15.7)	1843 (20.5)	3255 (14.5)	<0.001	490 (14.5)	947 (14.1)	2662 (15.8)	0.002
Depression	217 (4.8)	438 (4.9)	746 (3.3)	<0.001	63 (1.9)	112 (1.7)	306 (1.8)	0.67
Dementia	113 (2.5)	289 (3.2)	597 (2.7)	0.013	35 (1.0)	78 (1.2)	167 (1.0)	0.52
Alcohol‐related illness	16 (0.4)	90 (1.0)	141 (0.6)	<0.001	13 (0.4)	27 (0.4)	58 (0.3)	0.79
Medication, %								
Insulin	2119 (47.2)	5920 (65.9)	2013 (9.0)	<0.001	422 (12.5)	968 (14.4)	1690 (10.0)	<0.001
Oral antidiabetic agent	1952 (43.5)	5470 (60.9)	4252 (18.9)	<0.001	688 (20.4)	1623 (24.1)	3350 (19.9)	<0.001
Statins	2396 (53.4)	3051 (34.0)	2252 (10.0)	<0.001	657 (19.5)	1429 (21.2)	3111 (18.5)	<0.001
Fibrate	713 (15.9)	982 (10.9)	656 (2.9)	<0.001	150 (4.5)	329 (4.9)	701 (4.2)	0.05
Aspirin	1133 (25.2)	3443 (38.3)	2944 (13.1)	<0.001	461 (13.7)	1048 (15.6)	2295 (13.6)	<0.001
NSAIDs	1419 (31.6)	3545 (39.5)	7801 (34.7)	<0.001	875 (26.0)	1714 (25.4)	4646 (27.6)	0.002
ACEI	723 (16.1)	2117 (23.6)	1789 (8.0)	<0.001	416 (12.4)	746 (11.1)	2240 (13.3)	<0.001
Hormone replacement therapy	122 (2.7)	259 (2.9)	848 (3.8)	0.001	88 (2.6)	128 (1.9)	417 (2.5)	0.02

PD, peritoneal dialysis; HD, hemodialysis; SD, standard deviation; chronic lung disease, chronic obstructive pulmonary disease and asthma; NSAIDs, nonsteroid anti‐inflammatory drugs; ACEI, angiotensin‐converting enzyme inhibitors.

In Table 2, in the age‐ and sex‐matched models, after adjusting for age, sex, comorbid clinical illnesses, and medications, the adjusted HR of some cancers in PD patients was higher than that in comparison patients, including hepatocellular carcinoma (1.58; 95% CI, 1.06–2.36), bladder cancer (13.85; 95% CI, 8.19–23.41), extra kidney and bladder/urinary tract cancer (26.75; 95% CI, 10.4–68.81), and thyroid cancer (2.86; 95% CI, 1.01–6.13). At the same time, the adjusted HR of some cancer diseases was also higher in HD patients than in comparison patients, including hepatocellular carcinoma (1.48; 95% CI, 1.08–2.02), kidney cancer (10.12; 95% CI, 2.38–43.0), bladder cancer (14.04; 95% CI, 8.66–22.76), extra kidney and bladder urinary tract cancer (22.86; 95% CI, 9.43–55.4), and thyroid cancer (2.69; 95% CI, 1.09–6.62). However, in Table 3, after adjustment for risk of death in the age‐ and sex‐matched models, the risks of hepatocellular carcinoma and thyroid cancer were no more significantly different in the PD patient than those in the comparison group.

**Table 2:** Age‐ and sex‐matched and propensity score‐matched multivariable‐adjusted Cox regression models hazard ratios of cancer among the hemodialysis, peritoneal dialysis, and nondialysis comparison cohorts during follow‐up

	Age and sex matched			Propensity score matched		
	PD(*N* = 4491)	HD(*N* = 8982)	Comparison(*N* = 22,455)	PD(*N* = 3369)	HD(*N* = 6738)	Comparison(*N* = 16,845)
Total cancer	200 (4.5)	441 (4.9)	813 (3.6)	111 (3.3)	222 (3.3)	388 (2.3)
	1.54 (1.30, 1.82)^a^	1.59 (1.39, 1.82)^a^	1	2.08 (1.68, 2.58)^a^	1.82 (1.54, 2.15)^a^	1
Lip, oral cavity, and pharynx cancer	7 (0.2)	26 (0.3)	69 (0.3)	4 (0.1)	18 (0.3)	21 (0.1)
	0.47 (0.21, 1.08)	0.82 (0.48, 1.39)	1	1.43 (0.49, 4.20)	2.75 (1.46, 5.19)^a^	1
Esophageal cancer	4 (0.1)	4 (0.04)	22 (0.1)	3 (0.1)	2 (0.03)	7 (0.04)
	1.09 (0.33, 3.57)	0.58 (0.19, 1.81)	1	3.19 (0.81, 12.59)	0.92 (0.19, 4.45)	1
Gastric cancer	7 (0.2)	19 (0.2)	57 (0.3)	3 (0.1)	4 (0.1)	20 (0.1)
	0.64 (0.28, 1.48)	0.91 (0.51, 1.62)	1	1.33 (0.39, 4.53)	0.68 (0.23, 2.02)	1
Colorectal cancer	22 (0.5)	61 (0.7)	141 (0.6)	12 (0.4)	29 (0.4)	84 (0.5)
	0.98 (0.60, 1.59)	1.28 (0.91, 1.82)	1	1.18 (0.64, 2.17)	1.14 (0.74, 1.74)	1
Hepatocellular carcinoma	35 (0.8)	89 (1.0)	129 (0.6)	13 (0.4)	22 (0.3)	36 (0.2)
	1.58 (1.06, 2.36)^a^	1.48 (1.08, 2.02)^a^	1	2.88 (1.52, 5.48)^a^	2.05 (1.20, 3.50)^a^	1
Gallbladder and bile ducts cancer	0 (0.0)	6 (0.1)	18 (0.1)	0 (0.0)	3 (0.04)	13 (0.1)
	NA	0.68 (0.24, 1.99)	1	NA	0.75 (0.21, 2.66)	1
Pancreatic cancer	0 (0.0)	5 (0.1)	17 (0.1)	0 (0.0)	6 (0.1)	9 (0.1)
	NA	0.83 (0.26, 2.59)	1	NA	1.92 (0.68, 5.40)	1
Retroperitoneum and peritoneum cancer	0 (0.0)	0 (0.0)	2 (0.01)	0 (0.0)	0 (0.0)	0 (0.0)
	NA	NA	1	NA	NA	1
Lung cancer	18 (0.4)	43 (0.5)	138 (0.6)	12 (0.4)	16 (0.2)	52 (0.3)
	0.84 (0.50, 1.41)	0.90 (0.62, 1.33)	1	1.88 (0.99, 3.54)	1.01 (0.57, 1.77)	1
Breast cancer (female)	19 (0.4)	32 (0.4)	92 (0.4)	11 (0.3)	16 (0.2)	56 (0.3)
	1.35 (0.77, 2.38)	0.92 (0.57, 1.48)	1	1.25 (0.65, 2.38)	0.84 (0.48, 1.46)	1
Cervical cancer	6 (0.1)	14 (0.2)	33 (0.2)	3 (0.1)	5 (0.1)	15 (0.1)
	0.80 (0.30, 2.11)	0.89 (0.42, 1.86)	1	1.29 (0.37, 4.45)	0.97 (0.35, 2.68)	1
Prostate cancer	3 (0.1)	8 (0.1)	37 (0.2)	2 (0.1)	3 (0.04)	34 (0.2)
	0.53 (0.16, 1.79)	0.72 (0.31, 1.65)	1	0.59 (0.14, 2.50)	0.32 (0.10, 1.06)	1
Kidney cancer	3 (0.1)	10 (0.1)	3 (0.01)	4 (0.1)	5 (0.1)	6 (0.04)
	3.83 (0.66, 22.16)	10.12 (2.38, 43.0)^a^	1	4.50 (1.26, 16.04)^a^	2.50 (0.76, 8.20)	1
Bladder cancer	51 (1.1)	83 (0.9)	26 (0.1)	26 (0.8)	70 (1.0)	13 (0.1)
	13.85 (8.19, 23.41)^a^	14.04 (8.66, 22.76)^a^	1	14.53 (7.44, 28.37)^a^	17.21 (9.50, 31.16)^a^	1
Extra kidney and bladder urinary tract cancer	22 (0.5)	30 (0.3)	7 (0.03)	13 (0.4)	20 (0.3)	5 (0.03)
	26.75 (10.40, 68.81)^a^	22.86 (9.43, 55.40)^a^	1	17.45 (6.20, 49.07)^a^	12.26 (4.59, 32.72)^a^	1
Brain cancer	0 (0.0)	6 (0.1)	7 (0.03)	0 (0.0)	0 (0.0)	0 (0.0)
	NA	1.92 (0.48, 7.60)	1	NA	NA	1
Thyroid cancer	8 (0.2)	14 (0.2)	13 (0.1)	8 (0.2)	12 (0.2)	14 (0.1)
	2.86 (1.01, 6.13)^a^	2.69 (1.09, 6.62)^a^	1	3.22 (1.34, 7.76)^a^	2.47 (1.14, 5.36)^a^	1
Lymphatic and hematopoietic cancer	3 (0.1)	13 (0.1)	31 (0.1)	0 (0.0)	5 (0.1)	16 (0.1)
	0.62 (0.18, 2.15)	1.21 (0.57, 2.54)	1	NA	1.02 (0.37, 2.80)	1

Data are presented as number of events (percentage of group with event) and adjusted HR (95% CI). Adjustments were made for age, sex, selected comorbidities, and medications.

HR, hazard ratio; CI, confidence interval; CRR, Fine and Gray competing‐risk regression; PD, peritoneal dialysis; HD, hemodialysis.

a *P* < 0.05.

**Table 3:** Age‐ and sex‐matched and propensity score‐matched multivariable‐adjusted competing‐risk regression models hazard ratios of cancer among the hemodialysis, peritoneal dialysis, and nondialysis comparison cohorts during follow‐up

	Age and sex matched			Propensity score matched		
	PD(*N* = 4491)	HD(*N* = 8982)	Comparison(*N* = 22,455)	PD(*N* = 3369)	HD(*N* = 6738)	Comparison (*N* = 16,845)
Total cancer	1.38 (1.11, 1.72)^a^	1.59 (1.32, 1.91)^a^	1	2.27 (1.84, 2.81)^a^	1.96 (1.66, 2.31)^a^	1
Lip, oral cavity, and pharynx cancer	0.27 (0.08, 1.02)	0.59 (0.29, 1.19)	1	1.46 (0.50, 4.29)	2.89 (1.56, 5.34)^a^	1
Esophageal cancer	0.30 (0.04, 2.58)	0.23 (0.03, 1.89)	1	3.41 (0.99‐11.64)	0.99 (0.22, 4.46)	1
Gastric cancer	0.52 (0.15, 1.85)	1.21 (0.56, 2.61)	1	1.44 (0.42, 4.95)	0.74 (0.26, 2.14)	1
Colorectal cancer	0.58 (0.29, 1.19)	1.02 (0.67, 1.58)	1	1.23 (0.66, 2.27)	1.23 (0.81, 1.87)	1
Hepatocellular carcinoma	1.59 (0.90, 2.81)	1.59 (1.01, 2.17)^a^	1	3.04 (1.61, 5.74)^a^	2.23 (1.31, 3.77)^a^	1
Gallbladder and bile ducts cancer	NA	0.38 (0.14, 1.08)	1	NA	0.84 (0.24, 2.89)	1
Pancreatic cancer	NA	NA	1	NA	2.09 (0.75, 5.85)	1
Retroperitoneum and peritoneum cancer	NA	NA	1	NA	NA	1
Lung cancer	0.28 (0.09, 1.05)	0.72 (0.38, 1.39)	1	1.98 (1.05, 3.76)^a^	1.10 (0.62, 1.95)	1
Breast cancer (female)	1.02 (0.57, 1.84)	0.82 (0.48, 1.38)	1	1.26 (0.66, 2.43)	0.86 (0.49, 1.50)	1
Cervical cancer	0.82 (0.30, 2.24)	0.57 (0.20, 1.62)	1	1.30 (0.38, 4.48)	1.00 (0.37, 2.73)	1
Prostate cancer	NA	0.43 (0.18, 1.01)	1	0.63 (0.15, 2.73)	0.36 (0.11, 1.22)	1
Kidney cancer	4.78 (0.59, 38.69)	4.14 (1.18, 14.56)^a^	1	4.53 (1.22, 16.86)^a^	2.57 (0.78, 8.48)	1
Bladder cancer	16.49 (8.05, 33.76)^a^	19.36 (9.38, 39.76)^a^	1	14.99 (7.64, 29.41)^a^	17.92 (9.87, 32.54)^a^	1
Extra kidney and bladder urinary tract cancer	23.91 (8.04, 71.06)^a^	23.43 (8.75, 62.69)^a^	1	17.64 (6.31, 49.35)^a^	12.63 (4.78, 33.37)^a^	1
Brain cancer	NA	5.43 (0.94, 31.38)	1	NA	NA	1
Thyroid cancer	2.68 (0.91, 7.89)	2.97 (1.11, 7.94)^a^	1	3.50 (1.50, 8.18)^a^	2.56 (1.18, 5.54)^a^	1
Lymphatic and hematopoietic cancer	0.92 (0.17, 5.04)	0.97 (0.25, 3.75)	1	NA	1.02 (0.37, 2.84)	1

Adjustments were made for age, sex, selected comorbidities, and medications. Data are presented as adjusted HR (95% CI).

HR, hazard ratio; CI, confidence interval; CRR, Fine and Gray competing‐risk regression; PD, peritoneal dialysis; HD, hemodialysis.

a *P* < 0.05.

In Table 2, in the propensity score‐matched model, the adjusted HR of some cancers in PD patients was higher than that in the comparison patients, including hepatocellular carcinoma (2.88; 95% CI, 1.52–5.48), kidney cancer (4.50; 95% CI, 1.26–16.04), bladder cancer (14.53; 95% CI, 7.44–28.37), extra kidney and bladder/urinary tract cancer (17.45; 95% CI, 6.20–49.07), and thyroid cancer (3.22; 95% CI, 1.34–7.76). At the same time, the adjusted HR of some cancer diseases was also higher in the HD patients than in the comparison patients, including lip, oral cavity, and pharyngeal cancers (2.75; 95% CI, 1.46–5.19), hepatocellular carcinoma (2.05; 95% CI, 1.20–3.50), bladder cancer (17.21; 95% CI, 9.50–31.16), extra kidney and bladder urinary tract cancers (12.26; 95% CI, 4.59–32.72), and thyroid cancer (2.47; 95% CI, 1.14–5.36). However, in Table 3, after adjustment for risk of death in the propensity score‐matched model, the risk of lung cancer became significantly higher in the PD patients.

**Figure 2:**
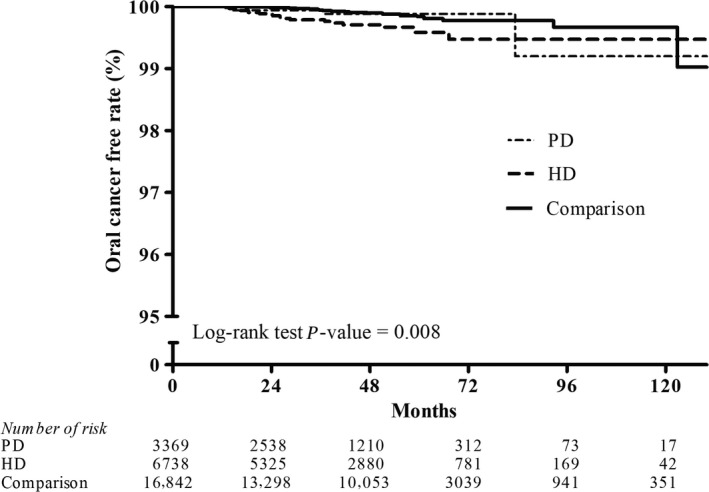
Kaplan–Meier curves of oral cancer disease‐free rate in HD, PD, and nondialysis comparison cohorts in propensity score‐matched models (PD vs. HD, *P *= 0.053; PD vs. comparison, *P *= 0.217; HD vs. comparison, *P *= 0.010; unadjusted association). HD, hemodialysis; PD, peritoneal dialysis.

**Figure 3:**
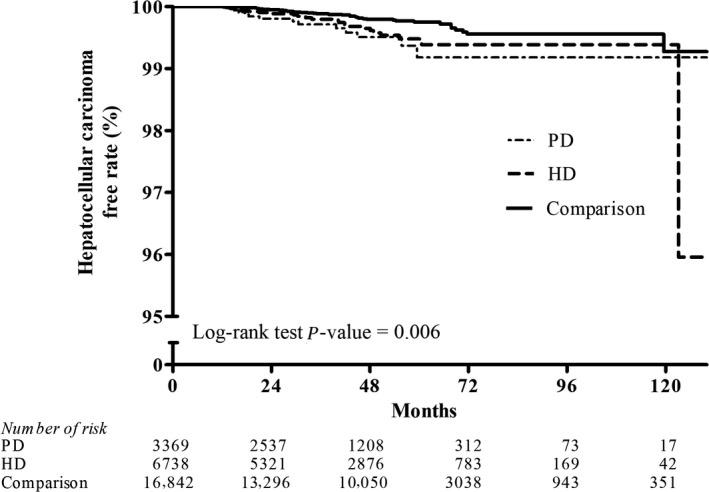
Kaplan–Meier curves of hepatocellular cancer disease‐free rate in HD, PD, and nondialysis comparison cohorts in propensity score‐matched models (PD vs. HD, *P *= 0.994; PD vs. comparison, *P *= 0.006; HD vs. comparison, *P* = 0.031; unadjusted association). HD, hemodialysis; PD, peritoneal dialysis.

**Figure 4:**
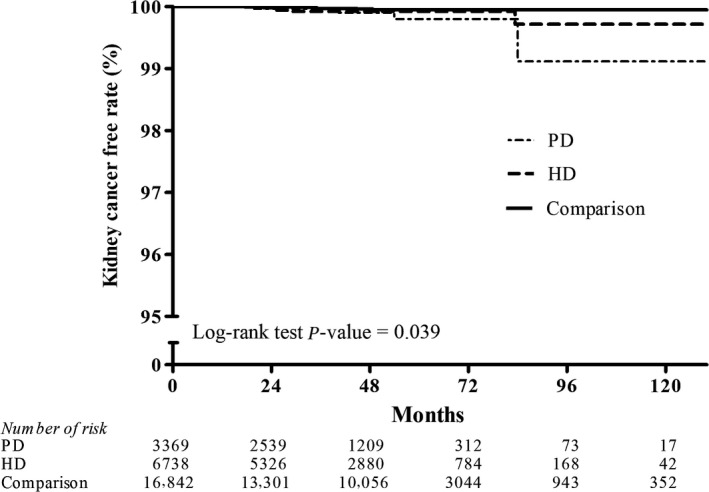
Kaplan–Meier curves of kidney cancer disease‐free rate in HD, PD, and nondialysis comparison cohorts in propensity score‐matched models (PD vs. HD, *P *= 0.921; PD vs. comparison, *P*=0.039; HD vs. comparison, *P *= 0.212; unadjusted association). HD, hemodialysis; PD, peritoneal dialysis.

**Figure 5:**
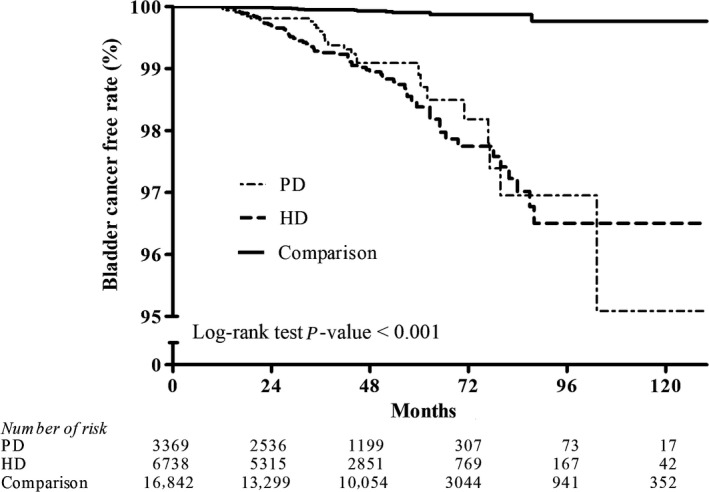
Kaplan–Meier curves of bladder cancer disease‐free rate in HD, PD, and nondialysis comparison cohorts in propensity score‐matched models (PD vs. HD, *P *< 0.001; PD vs. comparison, *P* < 0.001; HD vs. comparison, *P *< 0.001; unadjusted association). HD, hemodialysis , PD, peritoneal dialysis.

**Figure 6:**
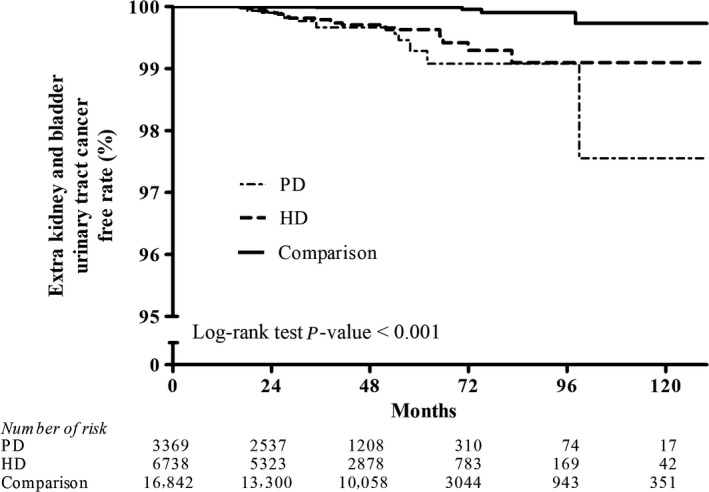
Kaplan–Meier curves of extra kidney and bladder urinary tract cancer disease‐free rate in HD, PD, and nondialysis comparison cohorts in propensity score‐matched models (PD vs. HD, *P *= 0.783; PD vs. comparison, *P *< 0.001; HD vs. comparison, *P *< 0.001; unadjusted association). HD, hemodialysis; PD, peritoneal dialysis.

**Figure 7:**
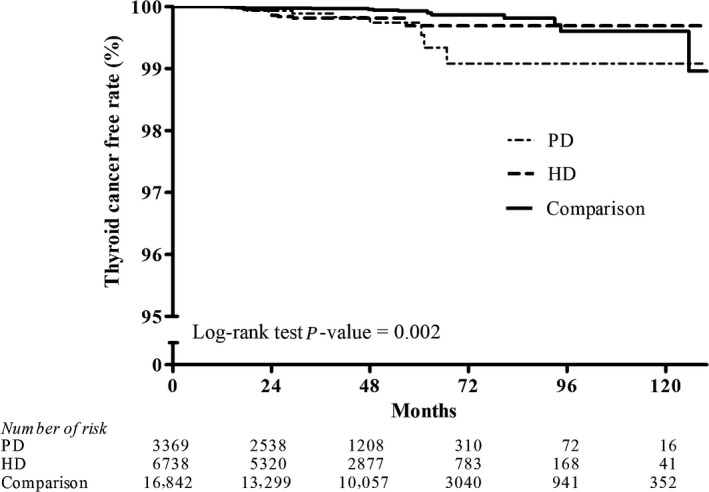
Kaplan–Meier curves of thyroid cancer disease‐free rate in HD, PD, and nondialysis comparison cohorts in propensity score‐matched models (PD vs. HD, *P *= 1.000; PD vs. comparison, *P *= 0.002; HD vs. comparison, *P *= 0.021; unadjusted association). HD, hemodialysis; PD, peritoneal dialysis.

Figures 2, 3, 4, 5, 6, 7 show Kaplan–Meier curves illustrating the disease‐free survival rate for lip, oral cavity, and pharyngeal cancer, hepatocellular carcinoma, kidney cancer, bladder cancer, extra kidney and bladder urinary tract cancer, and thyroid cancer in the HD, PD, and nondialysis comparison cohorts in propensity score‐matched models.

As one of the main purposes of this study was to compare the risk of development of cancer between HD and PD patients, we further compared the risk of different cancers between the two dialysis groups by using the HD patients as the reference group. As shown in Table 4, we found that the risk of cancer was not significantly different between the two dialysis groups.

**Table 4:** Age‐ and sex‐matched and propensity score‐matched multivariable‐adjusted Cox regression models hazard ratios of cancer among the hemodialysis and peritoneal dialysis cohorts during follow‐up

	Age and sex matched		Propensity score matched	
	PD(*N* = 4491)	HD(*N* = 8982)	PD(*N* = 3369)	HD(*N* = 6738)
Total cancer	0.87 (0.73, 1.05)	1	1.12 (0.89, 1.41)	1
Lip, oral cavity, and pharynx cancer	0.52 (0.22, 1.25)	1	0.50 (0.17, 1.47)	1
Esophageal cancer	1.51 (0.33, 6.94)	1	3.07 (0.51, 18.36)	1
Gastric cancer	0.61 (0.24, 1.53)	1	2.01 (0.44, 9.12)	1
Colorectal cancer	0.74 (0.44, 1.25)	1	0.98 (0.50, 1.93)	1
Hepatocellular carcinoma	0.96 (0.63, 1.46)	1	1.39 (0.70, 2.75)	1
Gallbladder and bile ducts cancer	NA	1	NA	1
Pancreatic cancer	NA	1	NA	1
Retroperitoneum and peritoneum cancer	NA	1	NA	1
Lung cancer	0.99 (0.55, 1.77)	1	1.87 (0.88, 3.96)	1
Breast cancer (female)	1.33 (0.69, 2.54)	1	1.52 (0.71, 3.28)	1
Cervical cancer	0.95 (0.33, 2.75)	1	1.32 (0.32, 5.54)	1
Prostate cancer	0.65 (0.16, 2.63)	1	2.18 (0.36, 13.24)	1
Kidney cancer	0.42 (0.10, 1.70)	1	1.87 (0.50, 7.00)	1
Bladder cancer	0.95 (0.66, 1.41)	1	0.84 (0.53, 1.31)	1
Extra kidney and bladder urinary tract cancer	1.18 (0.64, 2.18)	1	1.38 (0.69, 2.78)	1
Brain cancer	NA	1	NA	1
Thyroid cancer	0.94 (0.35, 2.50)	1	1.29 (0.53, 3.17)	1
Lymphatic and hematopoietic cancer	0.42 (0.11, 1.62)	1	NA	1

Data are presented as adjusted HR (95% CI). Adjustments were made for age, sex, selected comorbidities, and medications.

HR, hazard ratio; CI, confidence interval; CRR, Fine and Gray competing‐risk regression; PD, peritoneal dialysis; HD, hemodialysis.

Further validation analysis of the competing risk in the propensity score‐matched model also showed there was no significant difference between the two dialysis groups (Table 5).

**Table 5:** Age‐ and sex‐matched and propensity score‐matched multivariable‐adjusted competing‐risk regression models hazard ratios of cancer among the hemodialysis and peritoneal dialysis cohorts during follow‐up

	Age and sex matched		Propensity score matched	
	PD(*N* = 4491)	HD(*N* = 8982)	PD(*N* = 3369)	HD(*N* = 6738)
Total cancer	0.79 (0.62, 0.99)	1	1.11 (0.88, 1.39)	1
Lip, oral cavity, and pharynx cancer	0.42 (0.12, 1.49)	1	0.49 (0.16, 1.46)	1
Esophageal cancer	0.22 (0.01, 25.07)	1	3.06 (0.52, 18.12)	1
Gastric cancer	0.37 (0.10, 1.35)	1	1.98 (0.43, 9.11)	1
Colorectal cancer	0.54 (0.26, 1.11)	1	0.98 (0.48, 1.93)	1
Hepatocellular carcinoma	0.97 (0.53, 1.78)	1	1.35 (0.68, 2.69)	1
Gallbladder and bile ducts cancer	NA	1	NA	1
Pancreatic cancer	NA	1	NA	1
Retroperitoneum and peritoneum cancer	NA	1	NA	1
Lung cancer	0.49 (0.14, 1.70)	1	1.84 (0.88, 3.86)	1
Breast cancer (female)	1.03 (0.49, 2.20)	1	1.49 (0.69, 3.22)	1
Cervical cancer	1.37 (0.38, 4.87)	1	1.30 (0.31, 5.45)	1
Prostate cancer	NA	1	2.16 (0.36, 13.16)	1
Kidney cancer	0.70 (0.17, 2.88)	1	1.83 (0.48, 7.04)	1
Bladder cancer	0.84 (0.56, 1.24)	1	0.83 (0.53, 1.30)	1
Extra kidney and bladder urinary tract cancer	1.04 (0.53, 2.02)	1	1.40 (0.70, 2.80)	1
Brain cancer	NA	1	NA	1
Thyroid cancer	0.83 (0.27, 2.56)	1	1.28 (0.52, 3.12)	1
Lymphatic and hematopoietic cancer	0.63 (0.09, 4.49)	1	NA	1

Data are presented as adjusted HR (95% CI). Adjustments were made for age, sex, selected comorbidities, and medications.

HR, hazard ratio; CI, confidence interval; CRR, Fine and Gray competing‐risk regression; PD, peritoneal dialysis; HD, hemodialysis.

## Discussion

We conducted a large‐scale, retrospective cohort study using a nationwide database to investigate the cancer risk among HD, PD, and nondialysis patients. Our database enrolled almost all dialysis patients in the country and we used several different models to investigate this question. Generally, the results in these four different models were similar. In propensity score‐matched multivariable‐adjusted competing‐risk regression models, the results showed higher risks of hepatocellular carcinoma, bladder cancer, extra kidney/bladder urinary tract cancer, and thyroid cancer in both the HD and PD patients. The risks of lung and kidney cancers were only higher in the PD group, and the risks of lip, oral cavity, and pharyngeal cancers were only higher in the HD group. Because whether there are different cancer risks between HD and PD patients is an important issue, we also compared the risk of cancer between the two dialysis groups by using the HD patients as the reference group. We found that there is no significant difference in cancer risk between the two dialysis groups. To the best of our knowledge, this is the first study to compare cancer risks between HD and PD groups directly. In summary, we found that dialysis patients had a higher risk of certain types of cancers than patients in the non‐ESRD group. However, there was no significant difference in the cancer risk between the two dialysis groups when compared directly.

Some previous studies have investigated the association between cancer and ESRD 3, 4, 5, 6, 7, 8, 9, 10, 11, 12, 13, 14, 15, 16, 17, 18, 19, 20, 21. However, the findings of these studies are controversial and had some limitations. First, many of these studies included only a small number of subjects and the prevalence of some of the cancers was low. In addition, most studies only enrolled local area populations and their follow‐up time was relatively short. Second, patients who received PD therapy usually had better baseline characteristics and fewer comorbidities than patients receiving HD therapy, and therefore there might be selection bias if only age‐ and sex‐matched patients were used in the analysis. Despite this, none of these previous studies used another matched model to correct for this bias. Third, death may act as a competing risk for cancer, but previous studies did not use competing‐risk models to adjust for the risk of death. Fourth, many of these studies were not cohort studies and therefore a cause and effect type of relationship between the dialysis modalities and cancer could not be determined. Fifth, in some studies, there was a lack of adjustment for certain cancer risk factors, including medications and comorbidity, in multivariable analysis. Sixth, some cohort studies did not exclude nonincident ESRD patients and the actual incidence rate of cancer could not be known. Some of the studies also lacked nondialysis patients as a comparison group. Most importantly, we have reasonable grounds to suspect that HD and PD patients may have different cancer risks, especially with regard to cancer of the intra‐abdominal organs, as PD patients have a risk of chronic peritoneal inflammation, while HD patients have an increased risk of hepatitis B and C, as well as of PUD. However, data about differences in cancer risk among HD and PD patients are still limited. Therefore, our study aimed to compare the cancer risk between these two dialysis groups directly.

The mechanism by which ESRD may influence the development of cancer is still not well understood and a multifactorial etiology is believed to be likely. There are some possible mechanisms as to why dialysis patients might have an increased risk of developing cancer. First, some studies show increased DNA damage in ESRD patients as a result of impaired DNA repair, which may result in tumor formation 40, 41. In addition, antioxidant capacity is reduced in uremic patients, which may also lead to DNA damage because of an increase in reactive oxygen species 42. Second, release of cytokines during the dialysis procedure due to bioincompatibility of the dialysis membrane has been suggested to predispose to malignancy 43. Third, ESRD patients suffer from an accumulation of carcinogenic agents because of a reduced renal elimination. Fourth, chronic infections or inflammation status in ESRD patients may accelerate malignant transformations and tumor formations 44. Finally, there is also evidence for immunity impairment in CKD patients, which can become worse after dialysis 45.

There could also be a different etiology for the various cancers. There are some possible reasons for higher risk of kidney and urinary tract cancers in the dialysis group. First, certain causal factors link kidney damage and tumorigenesis of the kidney and urinary tract. For example, plants containing aristolochic acid are frequently used in Asia 46. Aristolochic acid is a nephrotoxic agent and can also induce urinary tract cancer, and its use is one possible reason for the higher risk of urinary tract cancer in Asian ESRD patients. Another possibility is the use of analgesic agents. Analgesic abuse can induce kidney damage and is also associated with transitional cell carcinomas of the renal pelvis, ureters, and bladder 19, 47. In addition, renal stones, obstructive nephropathy, and polycystic kidney disease are risk factors for both ESRD and RCC or TCC 48, 49. Second, ESRD itself causes development of renal cysts, with the subsequent occurrence of renal cell carcinoma 50. Third, some immunosuppressive or cytotoxic therapies for glomerulonephritis or vasculitis that are used to treat CKD patients before dialysis may also increase the risk of bladder cancer 48. Finally, the presence of urinary stasis, chronic bladder irritation, chronic infection, a decreased urinary washout effect, and atrophic involution of the bladder are other possible factors predisposing to the development of transitional cell carcinomas of the urinary tract 51.

A higher prevalence of chronic hepatitis in ESRD patients has been reported 52. It is likely that chronic hepatitis may be involved in the development of liver cancer in dialysis patients. The reason why oropharyngeal, thyroid, and lung cancer risks were higher in the dialysis patients is still unknown and further investigation is required to confirm this finding.

There are some limitations in this study. First, the diagnoses of comorbidities relied on administrative claims data, which could have been misclassified. However, previous epidemiological database studies that also used the NHIRD database have proven it has acceptable quality, and we also validated the ESRD and cancer codes by chart review 31, 32, 33. Second, certain personal information such as BMI, smoking, and alcoholism were not available in this database, and these factors may be important in the occurrence of cancer events. For example, alcoholism is a well‐known risk factor for hepatocellular carcinoma. To resolve this problem, we adjusted for alcohol‐related illness (571.0–571.3), which is highly associated with alcoholism status. We also adjusted for COPD disease, not only because this disease is one possible risk factor for lung cancer, but also because COPD is highly associated with smoking status 53. Moreover, we used the obesity code (278.0) in the analysis instead of BMI. Third, the lead‐time bias is one of the limitations of our study. The increased frequency of medical visits and laboratory and imaging tests definitely increases the rate of diagnosis of cancer. In Taiwan, regular chest radiography is performed for dialysis patients at about 6‐monthly intervals usually. In some hospitals, dialysis patients also undergo ultrasound of the kidney every year. Patients undergo a thyroid/parathyroid ultrasound if hyperparathyroidism is noted, but not routinely. If patients have chronic hepatitis B or C, the nephrologist will usually suggest that dialysis patients undergo a regular abdominal ultrasound about every 6 months. On the other hand, abdominal CT is not a routine test, but depends on clinical indication. Generally, dialysis patients will undergo more frequent chest radiography, renal ultrasound, abdominal ultrasound, and thyroid/parathyroid ultrasound compared to non‐ESRD patients. Thus, the higher incidence of hepatocellular carcinoma, bladder cancer, and extra kidney/bladder urinary tract cancer in both PD and HD patients may be due to their more frequent diagnostic tests. Nevertheless, this cannot explain why the risks of lung and kidney cancers were only higher in the PD or HD group. Besides, it may be expected that the incidence of gallbladder and bile duct cancer, and pancreatic cancer would be higher in the dialysis group due to higher frequency of chest radiography and abdominal ultrasound. However, in our study the incidence of the gallbladder and bile duct cancer and pancreatic cancer were not significantly different from that in the non‐ESRD group. Therefore, we think that, although the more frequent medical visits and laboratory and image surveys increase the rate of diagnosis of cancer, they cannot totally explain the study findings.

In conclusion, our study found that the dialysis patients had a higher risk of some specific cancers. We suggest that patients undergoing dialysis should receive regular cancer assessments. In addition, more attention should be paid to specific types of cancer in these patients.

## Conflict of Interest

The authors have indicated no financial conflicts of interest.
